# Essential Newborn Care Virtual Simulations for Skills Retention in Newborn Care

**DOI:** 10.1001/jamanetworkopen.2024.60565

**Published:** 2025-02-20

**Authors:** Rachel A. Umoren, Chinyere Ezeaka, Sara K. Berkelhamer, Daniel S. Hippe, Ime E. Asangansi, Matthew W. Cook, Iretiola B. Fajolu, Olubukola Olawuyi, Christianah Adeboboye, Oluwadamilola O. Ekhalufoh, Omolola S. Fashola, John Feltner, Joseph D. Fisher, Jasmine M. James, Olukemi M. Imoukhuede, Nahee Park, Victoria Quach, Amanda K. Stiffler, Cyril M. Engmann

**Affiliations:** 1 Department of Pediatrics, University of Washington, Seattle; 2 Department of Paediatrics, University of Lagos, Lagos, Nigeria; 3 Fred Hutchinson Cancer Center, Seattle, Washington; 4 eHealth4everyone, Abuja, Nigeria; 5 Lagos University Teaching Hospital, Lagos, Nigeria

## Abstract

**Importance:**

Newborn mortality accounts for approximately 47% of all mortality of children under the age of 5 years. Virtual simulation may be a viable approach to support retention of essential newborn care knowledge and skills among health care professionals in low- and middle-income countries.

**Objective:**

To evaluate the association between mobile virtual simulation using Virtual Essential Newborn Care (vENC) and knowledge and skills retention in early newborn care in low-resource settings and to propose a frequency of virtual simulation use for among health care professionals who care for newborns in low-resource settings.

**Design, Setting, and Participants:**

This cohort study was conducted at 23 primary, secondary, and tertiary health care facilities in Lagos, Nigeria, for 6 months between December 1, 2022, and June 30, 2023. Participants included nurses and midwives who participated in deliveries and provided newborn care. Potential participants who attended a Helping Babies Breathe or Essential Newborn Care (ENC) course within the past 1 year were excluded.

**Exposures:**

All participants received in-person training using the World Health Organization ENC 1 and ENC 2 curricula along with virtual simulation practice at variable recommended frequencies for 6 months after course completion.

**Main Outcomes and Measures:**

Primary outcomes included assessments of bag-valve-mask (BVM) ventilation skills, and performance on ENC 1 and ENC 2 case A and B scenarios conducted by trained research assistants before, immediately after, and 6 months after the in-person course. All scores ranged from 0% to 100%, with higher scores indicating better performance.

**Results:**

Of 70 enrolled participants (67 of 69 [97%] female), 62 (89%) completed the 6-month follow-up. Immediate posttraining performance (median [IQR] scores: BVM ventilation skills, 93% [86%-100%]; ENC 1 case scenario A, 72% [61%-78%]; ENC 1 case scenario B, 76% [68%-88%]; ENC 2 case scenario A, 80% [73%-87%]; and ENC 2 case scenario B, 88% [70%-95%]) improved compared with pretraining performance for all skill assessments (median [IQR] scores: BVM ventilation skills, 57% [29%-64%]; ENC 1 case scenario A, 39% [28%-50%]); ENC 2 case scenario A, 33% [20%-45%]) (all *P* < .001). There were further gains in performance at the 6-month follow-up assessment for BVM ventilation (median [IQR], 100% [86%-100%]; *P* = .04) and the ENC1 and ENC2 assessments by case scenario (case scenario A: ENC 1 median [IQR] score, 78% [72%-83%]; *P* = .001 and ENC 2 median [IQR] score, 87% [80%-93%]; *P* = .008; and case scenario B: ENC 1 median [IQR] score, 88% [76%-92%]; *P* = .009 and ENC 2 median [IQR] score, 93% [80%-100%]; *P* = .004) relative to the immediate postcourse assessment scores.

**Conclusions and Relevance:**

Findings of this cohort study suggest that the app-based simulations may be effective in supporting the retention of knowledge and skills following ENC training and may contribute to further performance gains for health care professionals in low- and middle-income countries. More clinical and implementation research is needed to explore the impact of virtual simulations on health professionals’ clinical practices and neonatal outcomes.

## Key Points

**Question:**

Is the use of mobile virtual simulation associated with retention of health professionals’ knowledge and skills following training in essential newborn care in low-resource settings?

**Findings:**

This cohort study involving 70 nurses and midwives in Nigeria found that monthly mobile virtual simulations were associated with improved retention of knowledge and skills in neonatal resuscitation and newborn care.

**Meaning:**

These results suggest that health care professionals may optimize knowledge and skill retention related to early newborn care in low-resource settings with monthly practice using mobile virtual simulation.

## Teaser

This cohort study investigates whether the use of mobile virtual simulations after receipt of in-person training in essential newborn care is associated with retention of knowledge and skills among nurses and midwives in low-resource settings.

## Introduction

A child’s risk of death is highest during the neonatal period or first 28 days of postnatal life worldwide.^1^ One-third of these neonatal deaths occur on the first day alone, and three-quarters in the first week.^2^ The first week of life is therefore a critical period for targeted interventions to improve newborn outcomes. The main contributors to neonatal mortality are prematurity, intrapartum asphyxia, and infections.^3^ Low- and middle-income countries are impacted by greatest burden of neonatal mortality due to resource limitations and poor rates of antenatal care access.^4,5,6^ These factors are compounded by the shortage of health professionals with the skills to care for newborns.^3,7^

Training in essential newborn care often occurs in the setting of regional in-person training of administrative and in-service health professionals. These individuals then teach others in their facilities through a cascading training approach called “train the trainers.”^8^ However, other strategies have been proposed, including videos and internet-based training modules with or without remote facilitation.^9,10^ With increasing internet and mobile phone access, digital training options provide greater access to training materials than in-person training alone. However, opportunities for low-dose, high-frequency practice after initial training, while strongly recommended, have remained limited to manikin-based practice in local facilities, with peer or facilitator mentoring when available.^11,12,13^

**Figure 1.**
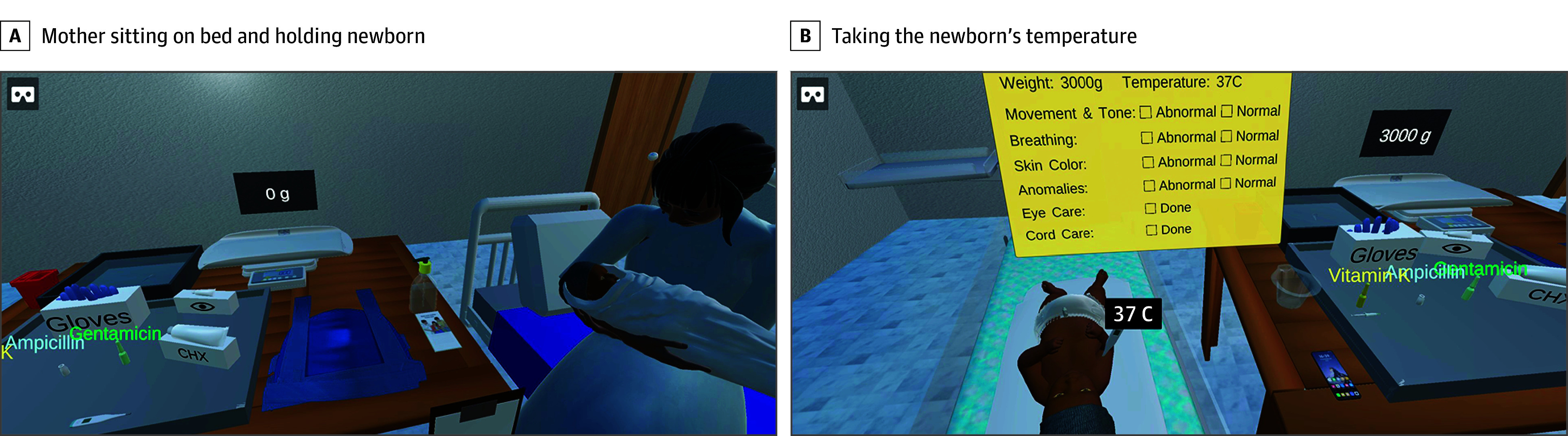
Virtual Essential Newborn Care Virtual Simulations

In 2018, pioneering work to leverage virtual simulations for health care professionals to practice neonatal resuscitation skills in a low-resource setting was conducted in Nigeria and Kenya using an app called electronic Helping Babies Breathe (eHBB).^14,15^ Similar to other digital interventions, virtual simulations were found to be acceptable and feasible in supporting knowledge and skills retention.^14,15,16^ However, the eHBB scenarios were limited to neonatal resuscitation and did not incorporate early newborn care. In addition, scenarios were based on the second edition of the HBB curriculum, which was later updated to align with the 2023 guidelines from the World Health Organization (WHO) with the introduction of the second edition WHO Essential Newborn Care (ENC) course.^17^ This led to the need for new virtual simulation scenarios to cover neonatal resuscitation and early newborn care. The Virtual Essential Newborn Care (vENC) simulations were then developed to provide practice opportunities and promote skills retention as a low-dose, high-frequency strategy for health professionals already trained in ENC. With the vENC virtual simulation on a smartphone or tablet, health professionals use their eyes or hands to interact with a 3-dimensional or virtual reality (VR) experience^18^ (Figure 1).

Each virtual simulation begins with preparation for the birth. Learners then perform the steps of essential newborn care, including immediate skin-to-skin care during the “golden hour,” obtaining weight and temperature, and providing eye care, umbilical cord care, and vitamin K. After performing an examination and classifying the infant by acuity and care needs as green, yellow, or red, the learners can choose to discharge the patient (green); provide additional supportive care, such as cup feeding, continuous skin-to-skin care, and temperature and weight monitoring (yellow); or refer the infant to a higher level of care (red). Communication for respectful maternal and newborn care is modeled throughout the virtual simulations along with infection prevention. Learners receive step-by-step guidance, automated feedback, and a final score based on their performance on approximately 100 within-scenario actions and use of hints. The app is easy to download, even on slower internet connections, and can be used offline. The objectives of this study were to evaluate the educational efficacy of the simulations among health care professionals who care for newborns in low-resource settings using mobile virtual simulations, and to propose a frequency of app use for retention of knowledge and skills.

## Methods

### Study Setting and Participants

This cohort study was conducted in Lagos, Nigeria, between December 1, 2022, and June 30, 2023. Study participants were recruited from 23 urban health care facilities providing maternal and newborn services. The facilities consisted of 14 (61%) primary health care facilities, 7 (30%) secondary health care facilities, and 2 (9%) tertiary health care facilities. Of these facilities, 21 (91%) were government-run and 2 (9%) were private facilities. All facilities had basic infrastructure (water from taps, electricity from grids, and roads leading to the facility). Many also offered ambulance services. We followed the Strengthening the Reporting of Observational Studies in Epidemiology (STROBE) reporting guideline for cohort studies.^19^ Ethics approval was obtained from the University of Lagos College of Medical Sciences and the Institutional Review Board at the University of Washington.

The health care system and staffing in Nigeria vary between primary, secondary, and tertiary facilities. Primary health care facilities are often staffed by midwives and nurses, who handle most newborn care. General physicians are not consistently available across all levels of care, particularly in rural or remote areas.^20^ Newborn care in Nigeria is also stratified, with essential newborn care being available at all facilities, and comprehensive care for sick newborns primarily offered at secondary or tertiary facilities.^21^ Overall, Nigeria is a lower middle–income country with a population of 230.2 million (2024), has limited resources devoted to newborn care, and has a high neonatal mortality rate.^22,23^ Study participants consisted of nurses and midwives assigned to labor and delivery and newborn care units.

### Inclusion and Exclusion Criteria

Nurses and midwives who participated in deliveries, provided newborn care, and provided study consent were included in this study. We excluded individuals who attended an HBB or ENC course in the 12 months preceding the study and individuals who did not provide essential newborn care as part of their duties or would be unavailable or unwilling to participate in follow-up study activities through the 6-month follow-up period.

### Recruitment, Consent, Enrollment

Study coordinators or research assistants (O.O. and C.A.) requested contact numbers, units, and wards of potential participants from head nurses at the identified facilities. Research assistants (O.O. and C.A.) contacted individuals to determine eligibility and obtained consent. Recruitment and in-person training were conducted concurrently at the University of Lagos, Nigeria, in December 2022, with follow-up continuing until June 2023.

Participants were enrolled and assigned a study identification number prior to the ENC course by local study coordinators. Upon enrollment, participants also took a demographic survey. After the 2-day in-person ENC 1 and ENC 2 courses, participants received a 20-minute orientation on how to use the app on the study phone with a low-cost VR headset. There were 6 recommended practice levels (in addition to no recommendation), a combination of a practice dose of 2, 4, or 6 scenarios, and a frequency of every 4 weeks or every 2 weeks (2, 4, 6, 8, or 12 scenarios per month). The primary purpose of the practice recommendations was to encourage a wide range of app use across the participants to facilitate subsequent analyses to assess the association of the amount of practice with testing performance. Participants were told that they could practice more frequently than their recommended number of scenarios if desired, and minimum recommendations were not enforced. eTable 1 in Supplement 1 shows the study procedures.

### ENC Training Module Overview

ENC 1 and 2 are training modules that align the educational strategies and content from the Helping Babies Survive programs with WHO recommendations for newborn care. ENC 1 focuses on care given during the first 60 minutes after birth, while ENC 2 addresses the care given after the first hour to the ensuing hours and days. Each ENC modules contain 2 case scenarios: A and B. Case scenario A in both ENC 1 and ENC 2 is about managing healthy newborns, with a focus on providing routine newborn care. Case scenario B in both ENC 1 and ENC 2 focuses on more complicated algorithms for newborn care and resuscitation. All ENC materials can be found on the WHO website^17^ and as an online teaching platform developed by the American Academy of Pediatrics and Laerdal Global Health called Helping Mothers and Babies Survive.^24^

### ENC Course Structure

To ensure that all participants received the same initial ENC training and baseline assessments using the new WHO ENC course second edition, a 2-day in-person ENC 1 and ENC 2 course was conducted by experienced facilitators (R.A.U., C.E., I.B.F., O.O., C.A., O.O.E., and O.M.I.) using the WHO ENC course second edition training materials.^17^ Health care professionals from primary, secondary, and tertiary facilities in Nigeria who had a direct clinical role related to newborn care were recruited to participate.

### Assessments Before and After the Training Course

Pretraining and posttraining assessments consisted of knowledge tests (knowledge check), direct observations on bag-valve-mask (BVM) ventilation skills, and performance on 4 case scenarios measuring cognitive and psychomotor skills. Standardized knowledge and skills assessments were conducted by trained research staff (R.A.U., C.E., I.B.F., O.O., C.A., O.O.E., and O.M.I.). The ENC 1 knowledge check (20 multiple-choice questions), ENC 2 knowledge check (25 multiple-choice question), and a 14-item BVM ventilation skills check were conducted before and after the course along with the ENC 1 and ENC 2 case scenario A. ENC 1 case scenario A is a 15-item checklist on preparation for delivery and initial steps of resuscitation. ENC 2 case scenario A is a 15-item checklist on care for a full-term infant 1 hour after birth. In addition, the assessment after the course included ENC 1 case scenario B, a 23-item checklist scenario featuring a newborn requiring prolonged newborn resuscitation, and ENC 2 case scenario B, a 20-item checklist comprising care of a preterm infant prior to referral for advanced care. The standard ENC course multiple-choice questions and checklists are available online.^17,24^ All ranged from 0 to 100, with higher numbers indicating better performance. A demographic survey was completed by each participant.

### Posttraining Course Interventions and Follow-Up

Participants were encouraged to practice virtual simulations and to engage in standard BVM ventilation skills practice with a manikin in their facility. Participants received monthly text message reminders to practice and monthly internet data (2 GB) to sync app user data to the database. Participants were not discouraged from practicing more than recommended, and practice recommendations were not otherwise enforced. Posttraining assessments were repeated 6 months after the course. Sharing of high scores through screenshots by participants was not part of the study design but often occurred spontaneously within social media groups set up by facilitators for information sharing and questions. A follow-up survey on user experience was also completed at that time.

### Data Collection

Data were collected in person by study staff who had completed an ENC master trainer course led by experienced ENC master trainers (R.A.U. and C.E.). Staff used paper forms and the RedCap mobile app for offline data collection. The vENC app separately tracked simulation access and use through uploads to an online database built on a DHIS2 (District Health Information Software 2) open-source platform. The simulation exposure was quantified using the app’s synced data and saved data on phones and a 6-month follow-up survey that included self-reported use (with options of never, a few times, monthly, weekly, every shift).

In addition to the standardized checklists, an advanced neonatal manikin (NeoNatalie Live; Laerdal) was used to carefully monitor ventilation time, a critical aspect of neonatal resuscitation that refers to the duration during which positive pressure ventilation is administered. The manikin offers high-fidelity feedback on key parameters, such as ventilation pressures and volumes.^25^ This neonatal manikin was used for data collection and automated performance-based feedback during the BVM ventilation and ENC 1 case scenario B assessments, which included a requirement for BVM ventilation. In both ENC 1 and ENC 2, case scenario B is considered more challenging than case scenario A; thus, while all other assessments were administered before and after the course, case scenario B assessments were administered only after the course to assure novel exposure.

### Sample Size

The goal was to enroll 70 participants to accommodate a dropout rate of 20% across 6 months to provide analyzable data from at least 56 participants in all analyses. For case scenarios and BVM ventilation skill assessments, a sample size of 56 would provide greater than 82% power to detect a sustained improvement in performance from before virtual simulation training to 6 months after if the true absolute mean change in scores was 10%. These calculations were performed using G*Power, version 3.1.9.2 based on the Wilcoxon signed rank test with 2-sided α = .05 and worst-case distributional assumptions. Additional assumed parameter values included SD scores of 20% and within-subject correlation between scores of 0.3, estimated from the eHBB study.^14^

### Statistical Analysis

Continuous variables were summarized as using the mean (SD) or median (IQR). Categorical variables were summarized as counts (percentages). Performance scores measured before the course, immediately after the course, and 6 months after the course were compared between time points using the Wilcoxon signed rank test. Item-level performance on the knowledge and skills assessments (each scored as correct vs incorrect) was compared between time points using the sign test. Overall performance scores were compared between demographic groups using the Wilcoxon rank sum test or the Kruskal-Wallis test. Spearman rank correlation was used to summarize associations between logged app activity (number of scenarios and total time on the app) and self-reported app activity. Spearman rank correlation was used to evaluate associations of logged and self-reported measures of app activity with performance on knowledge and skills assessments at 6 months after training. There were some missing data, most notably due to dropout on the 6-month assessment. Complete-case analysis was used throughout, in which participants were excluded from a summary or analysis if any required values were missing. All statistical calculations were performed using R, version 4.0 (R Project for Statistical Computing). Hypothesis tests were 2-sided, with statistical significance defined as *P* < .05. Most tests were not adjusted for the number of comparisons made to avoid a large loss of statistical power. However, *P* values from the item-level tests (14 to 25 items per assessment multiplied by 7 assessments) were adjusted using the Benjamini-Hochberg method^26^ to better balance the risk of false-positive and false-negative findings.

## Results

A total of 70 health professionals (67 of 69 [97%] female and 2 [3%] male) from 23 facilities (14 primary, 7 secondary, and 2 tertiary health facilities) were enrolled in the study. All participants attended both days of the in-person ENC training course. Of the 70 nurses and midwives who participated in the in-person training, 62 (89%) completed the 6-month follow-up (Table). Most participants (44 [63%]) had less than 10 years of experience. Nearly half of participants (29 of 68 [43%]) had prior HBB of ENC training more than 1 year before study participation.

**Table.** Demographic Characteristics of Study Participants

Characteristic	Participants, No. (%) (n = 70)
Sex	
Female	67 (97)
Male	2 (3)
Not available, No.	1
Age range, y	
21-30	20 (29)
31-40	24 (34)
≥41	26 (37)
Profession	
Nurse	33 (47)
Midwife	37 (53)
Level of facility	
Primary	31 (44)
Secondary	19 (27)
Tertiary	20 (29)
Years of experience	
<5	10 (14)
5-10	34 (49)
11-20	14 (20)
>20	12 (17)
Prior training for HBB or ENC	
Yes	29 (43)
No	39 (57)
Not available, No.	2

Abbreviations: ENC, Essential Newborn Care; HBB, Helping Babies Breathe.

### Pretraining and Posttraining Neonatal Resuscitation and Care Knowledge and Skills Assessments

**Figure 2.**
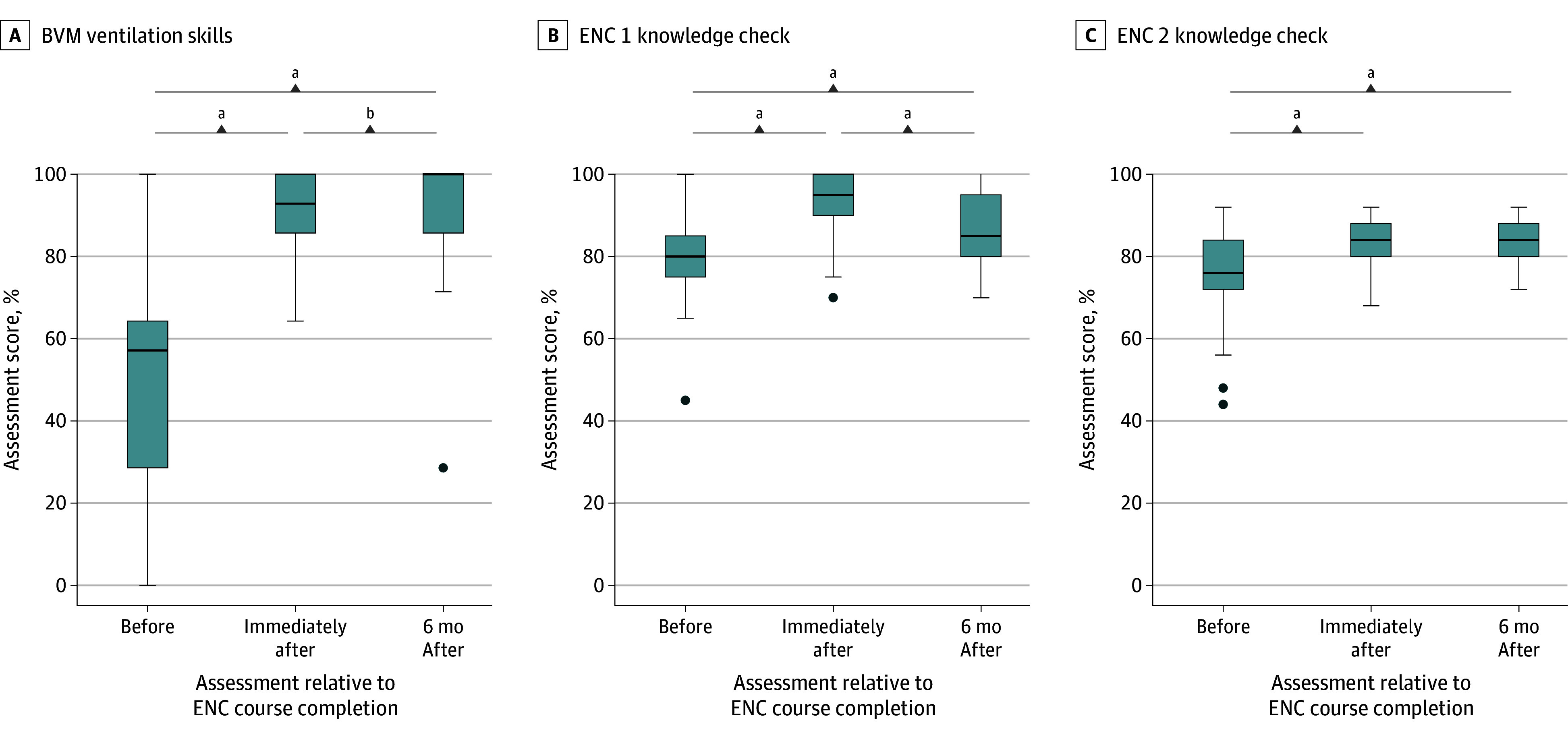
Bag-Valve-Mask (BVM) Ventilation Skills and Essential Newborn Care (ENC) Knowledge Check Assessments Over Time Assessments 6 months after attending the in-person ENC course included practice with the virtual ENC app. All scores ranged from 0% to 100%, with higher scores indicating better performance. Box represents 25th to 75th percentile (IQR); center line, median value; bottom whisker, smallest value within 1.5 × IQR of the lower quartile; top whisker, largest value within 1.5 × IQR of the upper quartile; and solid dots, any values not within 1.5 × IQR of the lower quartile or upper quartile. ^a^*P* < .001. ^b^*P* = .04.

**Figure 3.**
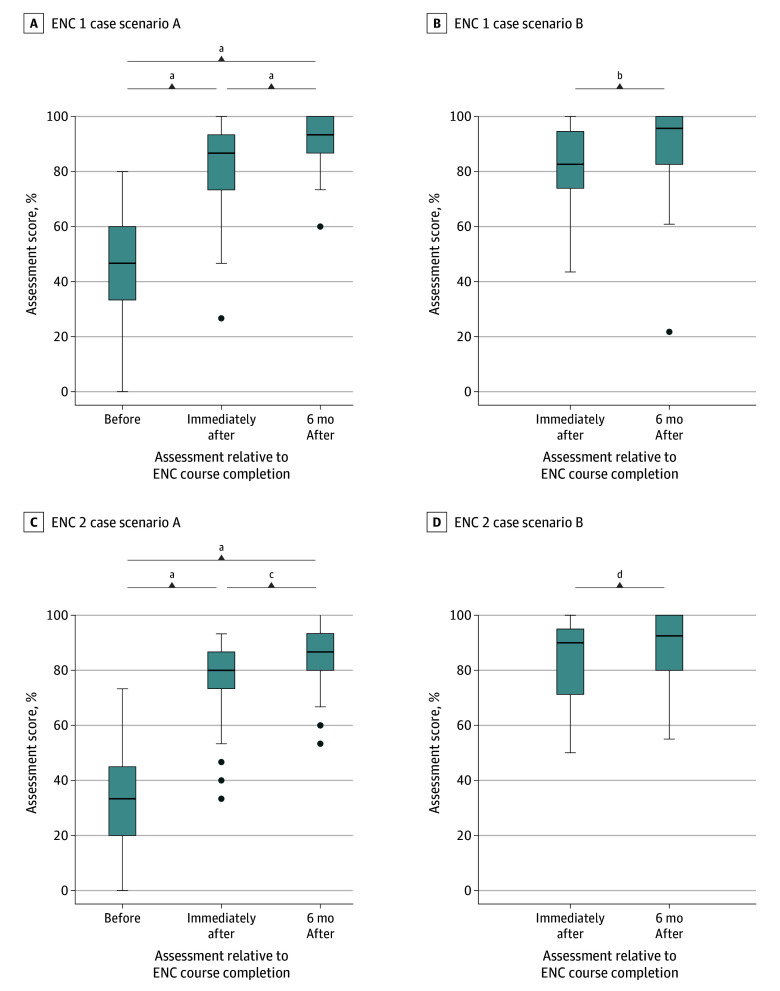
Essential Newborn Care (ENC) Case Scenario Scores Over Time Assessments 6 months after attending the in-person ENC course included practice with the virtual ENC app. All scores ranged from 0% to 100%, with higher scores indicating better performance. Box represents 25th to 75th percentile (IQR); center line, median value; bottom whisker, smallest value within 1.5 × IQR of the lower quartile; top whisker, largest value within 1.5 × IQR of the upper quartile; and solid dots, any values not within 1.5 × IQR of the lower quartile or upper quartile. ^a^*P* = .001. ^b^*P* = .007. ^c^*P* = .008. ^d^*P* = .004.

Neonatal resuscitation and neonatal care knowledge and skills assessments were conducted before the course, immediately after the course, and 6 months after the in-person course. Prior to training, ENC 1 and ENC 2 knowledge check scores were above 70% (median, 76%-80% [lower quartile, 72%-75%; upper quartile, 84%-85%]), while practical skills (BVM ventilation and case scenario) scores were lower (median, 33%-57% [lower quartile, 20%-33%; upper quartile, 45%-64%) (Figures 2 and 3).

The median (IQR) immediate posttraining scores (BVM ventilation, 93% [86%-100%]; ENC 1 knowledge check, 95% [90%-100%]; ENC 2 knowledge check, 84% [80%-88%]; ENC 1 case scenario A, 72% [61%-78%]; ENC 1 case scenario B, 76% [68%-88%]; ENC 2 case scenario A, 80% [73%-87%]; and ENC 2 case scenario B, 88% [70%-95%]) were significantly higher than the median (IQR) pretraining scores for all tests (BVM ventilation, 57% [29%-64%]; ENC 1 knowledge check, 80% [75%-85%]; ENC 2 knowledge check, 76% [72%-84%]; ENC 1 case scenario A, 39% [28%-50%]; and ENC 2 case scenario A, 33% [20%-45%]) (all *P* < .001), indicating better performance (Figures 2 and 3). There were further significant gains in performance at the 6-month follow-up assessment on the BVM ventilation (median [IQR], 100% [86%-100%]; *P* = .04) and the ENC 1 and ENC 2 assessments by case scenario (case scenario A: ENC 1 median [IQR] score, 78% [72%-83%]; *P* = .001 and ENC 2 median [IQR] score, 87% [80%-93%]; *P* = .008; and case scenario B ENC 1 median [IQR] score, 88% [76%-92%]; *P* = .007 and ENC 2 median [IQR] score, 93% [80%-100%]; *P* = .004) relative to the immediate postcourse assessment. By contrast, the ENC 1 knowledge check performance decreased from the immediate postcourse assessment to the 6-month postcourse assessment (median [IQR], 95% [90%-100%] vs 85% [80%-95%]; *P* < .001) although it remained higher than the precourse assessment (*P* < .001). The ENC2 knowledge check score was little changed at the 6-month postcourse assessment vs the immediate postcourse assessment (median [IQR], 84% [80%-88%] vs 84% [80%-88%]; both *P* = .51), and remained significantly higher than the precourse assessment score (*P* < .001). The ENC 1 knowledge check performance was higher before training with increasing years of experience (median [IQR], 70%-85% [66%-80%] to [80%-90%]; *P* = .02) (eTable 2 in Supplement 1), but these differences were no longer significant immediately after training (median [IQR], 95% [91%-95%] and 95% [90%-100%]; *P* = .80) or 6 months after training (median [IQR], 85%-90% [80%-91%] to [85%-95%]; *P* = .72).

### Performance of BVM Ventilation

Before training, median (IQR) BVM ventilation scores were 57% (29%-64%). After improving overall immediately after training, BVM ventilation skills assessed by checklist also showed an overall improvement in scores from the immediate postcourse assessment to the 6-month follow-up assessment instead of the expected decline (median [IQR], 93% [86%-100%] vs 100% [86%-100%]; *P* = .04) (Figure 2). Prior to training, median (IQR) BVM ventilation scores were higher with increasing facility level (primary, 43% [29%-61%]; secondary, 57% [29%-64%]; tertiary, 68% [57%-73%]; *P* = .008) and years of experience (eg, <5 years, 36% [9%-57%] vs 11-20 years, 64% [57%-71%]; *P* = .02) (eTable 2 in Supplement 1). However, these differences were no longer significant immediately after training (facility level median [IQR]: primary, 93% [86%-100%]; secondary, 93% [86%-100%]; tertiary, 93% [86%-100%]; *P* = .70; years of experience: <5, 86% [80%-98%] vs 11-20 years, 93% [88%-100%]; *P* = .53) or 6 months after training (median [IQR] for facility level: primary, 96% [86%-100%]; secondary, 96% [93%-100%]; tertiary, 100% [98%-100%]; *P* = .21; for years of experience <5, 100% [93%-100%] vs 11-20 years, 100% [91%-100%]; *P* = .66).

Analysis of the data collected using the neonatal manikin showed stable performance immediately after the course and at 6 months after the course, with no significant difference in median (IQR) percentage of ventilation time (83% [57%-100%] vs 84% [68%-100%]; *P* = .27). The number of participants providing appropriate ventilation rates (30-50 breaths per minute) at the assessment immediately after the course was 23 (49%) vs 14 (30%) at the 6-month follow-up (*P* = .06) among 47 participants assessed using the manikin at both time points. When a range of 40 to 60 breaths per minute was used, the number of participants providing appropriate ventilation was 22 of 47 (47%) at the assessment immediately after the course vs 26 of 47 (55%) at the 6-month follow-up (*P* = .54).

### Performance on Simple Scenarios (Case Scenario A)

Prior to the course, the median (IQR) case scenario A scores were 39% (28%-50%) for ENC 1 and 33% (20%-45%) for ENC2. After improving immediately after training, the median (IQR) case scenario A scores further improved from the immediate postcourse assessment to the 6-month assessment for ENC 1 (72% [61%-78%] vs 78% [72%-83%]; *P* = .001) and ENC 2 (80% [73%-87%] vs 87% [80%-93%]; *P* = .008), with notably improved performance on the following items: washed hands (40 of 57 [70%] vs 54 of 57 [95%]; adjusted *P* = .003); clamped or tied and cut the cord (48 of 57 [84%] vs 57 of 57 [100%]; adjusted *P* = .02); took steps to reduce risk of bleeding and monitor mother (16 of 56 [29%] vs 39 of 57 [68%]; adjusted *P* = .001); continued skin-to-skin care and monitored (44 of 57 [77%] vs 57 of 57 [100%]; adjusted *P* = .002); and indicated correct location for injection (42 of 56 [75%] vs 54 of 56 [96%]; adjusted *P* = .01). There were no differences by demographic characteristics in case scenario A performance before or after training.

### Performance on Complex Scenarios (Case Scenario B)

Median (IQR) scores improved overall on case scenario B assessments from immediately after the course to 6 months after the course for ENC 1 (76% [68%-88%] vs 93% [80%-100%]; *P* = .007) and ENC 2 (88% [70-95] vs 92% [80-100]; *P* = .004), with notably improved performance on the following items: clamped and cut cord and moved to area for ventilation (30 of 53 [57%] vs 46 of 53 [87%]; adjusted *P* = .03), cleared secretions from the mouth and nose as needed (41 of 53 [77%] vs 51 of 53 [94%]; *P* = .04), opened mouth slightly (34 of 53 [64%] vs 47 of 53 [89%]; adjusted *P* = .04), helper took steps to reduce risk of bleeding and monitored mother (4 of 53 [8%] vs 21 of 51 [41%]; adjusted *P* = .006), positioned infant with arms and legs flexed and head turned (41 of 54 [76%] vs 55 of 56 [98%]; adjusted *P* = .01), and rechecked temperature in 1 hour (43 of 56 [77%] vs 56 of 56 [100%]; adjusted *P* = .005). There were no differences by demographic characteristics in case scenario B performance.

### Participant Exposure to Intervention

**Figure 4.**
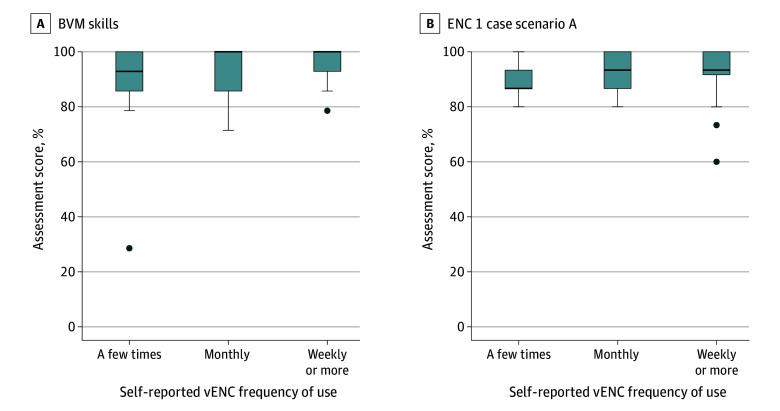
Performance vs Self-Reported Virtual Essential Newborn Care (vENC) Activity All scores ranged from 0% to 100%, with higher scores indicating better performance. At the 6-month assessment, self-reported vENC activity had a Spearman *r* of 0.30 (*P* = .02) with bag-valve-mask (BVM) ventilation skills and of 0.18 (*P* = .19) with ENC 1 case scenario A scores. The Spearman *r* for case scenario A scores correlated with the number of vENC scenarios performed during the study period was *r* = 0.24 (*P* = .09). Box represents 25th to 75th percentile (IQR); center line, median value; bottom whisker, smallest value within 1.5 × IQR of the lower quartile; top whisker, largest value within 1.5 × IQR of the upper quartile; and solid dots, any values not within 1.5 × IQR of the lower quartile or upper quartile.

In total, 60 of 70 participants (86%) had app activity recorded. Among these participants, the median (IQR) number of scenario sessions performed was 18 (4-38), with a median (IQR) of 1182 (193-2438) within-scenario actions. The median (IQR) total time spent in the app was 232 (40-493) minutes. Self-reported activity was available for 61 of 70 participants (87%). Most participants reported using the app at least weekly (34 [56%]), with the remainder using the app monthly (13 [21%]) or a few times during the study period (14 [23%]). The correlations between self-reported activity and the logged number of scenarios performed (Spearman *r* = 0.30; *P* = .02) or logged time in the app (Spearman *r* = 0.28; *P* = .03) were modest. BVM ventilation scores 6 months after the training were higher with increasing self-reported app use (Spearman *r* = 0.30; *P* = .02). There was no correlation between ENC 1 case scenario A scores and an increasing number of app-based scenarios performed (Spearman *r* = 0.24; *P* = .09) or an increasing number of within-scenario actions (Spearman *r* = 0.26; *P* = .05). Overall, these results suggest that at least monthly use of the app may be beneficial for maintaining performance after initial training (Figure 4). Monthly use corresponded to approximately 3 scenarios per month, with 200 within-scenario actions and 40 minutes use of the app, based on the median logged use of the participants who reported monthly use.

### Participant Feedback

Participant feedback indicated overall positive impressions of the app-based training intervention, as summarized in eTable 3 in Supplement 1. On the 6-month follow-up survey, with a Likert scale of 1 to 5 (range, 1 being strongly disagree and 5 being strongly agree), participants agreed or strongly agreed that the app was a convenient educational resource (50 of 61 [82%]), provided valuable feedback (51 of 61 [84%]), and was valuable for clinical practice (51 of 60 [85%]). Most participants would use the app again (46 of 58 [79%]) and would recommend it to their colleagues (47 of 55 [85%]). Positive ratings for ease of navigation were somewhat lower (36 of 60 [60%]), consistent with the common challenge reported of the navigation being slow at times when used on older devices (28 of 62 [45%]). Some participants also reported feeling nauseous or developing a headache when using the app in VR mode (17 of 62 [27%]), while all other challenges reported were relatively uncommon (eTable 3 in Supplement 1).

## Discussion

This cohort study is, to our knowledge, the first evaluation of the educational efficacy of mobile VR training on essential newborn care skills retention among health care professionals in a resource-scarce setting. In this study, virtual simulation training was effective in supporting the retention of knowledge and skills in essential newborn care out to 6 months after an in-person course. There was significantly improved performance 6 months after the course training from the immediate postcourse assessment for all 5 skill assessments, including BVM ventilation skills and ENC 1 and ENC 2 case scenarios A and B, which did not vary by participant experience or level of facility. These findings are comparable to a prior study on virtual simulations for neonatal resuscitation refresher training of health care professionals conducted in Nigeria and Kenya, in which a mobile virtual simulation, eHBB, based on the Helping Babies Breathe program was used to support knowledge and skills retention after an in-person course.^14^ In comparison to other digital interventions, the VR group pass rates on certain skill assessments featuring bag and mask ventilation (BVM ventilation skills and the Objective Structured Clinical Examination B) were maintained with no decline in performance from immediately after the course to the 6-month follow-up.^14^

With numerous studies demonstrating a decline in knowledge and skills following initial training,^8,27,28,29^ there is growing evidence and agreement that refresher training is necessary to maintain neonatal resuscitation skills. Videos on neonatal resuscitation have been evaluated for refresher training although the results have been mixed.^9,14,30^ Manikin-based refresher training is influenced by the availability of manikins, experienced facilitators or knowledgeable peers^13^ to provide feedback, and time for both facilitators and learners. However, more advanced manikins can provide individualized feedback through performance data accessible to learners through mobile devices.^31^ Similarly, refresher training may be limited by the time available to busy health care professionals; however, as virtual simulations do not require access to physical manikins and feedback is automated, participant engagement is less likely to be limited by these barriers but rather influenced by interest and engagement.^16,18^ For BVM ventilation skills, low-dose high-frequency manikin practice has been encouraged every shift. However, the recommended frequency of virtual refresher training had not been previously established. In our study, there was a high rate of weekly use, and BVM ventilation skills and ENC1 case scenario A performance were highest when the virtual simulations were performed at least monthly. Health professionals should be encouraged to use it at least weekly using the automated reminders built into the app to support deliberate practice until competence has been achieved, and monthly thereafter.

Digital interventions have been increasingly adopted in newborn care training, including virtual and remote simulation, leveraging their benefits for developing cognitive and communication skills.^9,10,32,33^ Virtual simulation supports learning through repetition, user engagement, and identity formation.^18,34^ While manikin-based practice is important, particularly for learners who are acquiring new psychomotor skills, such as BVM ventilation, the focus on bag and mask ventilation as a sole reason for manikin-based practice overlooks the potential for digital practice of other critical skills that are essential to improve the quality of newborn care. These skills include preparation for delivery, initial resuscitation steps, early breastfeeding support, skin-to-skin care, and danger sign identification and preventative care, such as eye care, cord care, and vitamin K.^35^ Virtual simulations also leverage auditory cues, such as the infant’s healthy or weak cry, grunting respirations, and visual feedback, through animations representing the infant’s posture and movements to support learning and recall.^18,34^ In addition, abnormalities of skin color, such as cyanosis and jaundice, can be represented through virtual simulations more easily than in manikin-based scenarios in which learners must be given this information by a facilitator.^18,34^

Access to virtual simulations in low-resource settings has been limited by app and platform features, including requirements for a personal computer and internet access. However, in areas of limited access to computers and poor internet connectivity, there is a need for apps that work offline and on mobile devices. Virtual simulations have been designed for use with VR headsets, but their limited battery life and cost decrease their utility in a low-resource setting. A key feature that supported the use of the app during this study was the relatively small file size, which facilitated downloads to smartphones through links and social media groups followed by offline use. The app automatically syncs to a database when connected to the internet, which can be used to generate reports for facilitators. Some participants shared screenshots of high scores on the social media group, which supported group engagement.

### Limitations

This study has some limitations. We recruited participants only from health care facilities in an urban setting. The study findings may not apply to health professionals who work in high-resource settings or in rural low-resource settings. There was a dropout or nonresponse rate of 11% to 14% by the 6-month follow-up, depending on the assessment, which could have biased some results. To ensure uniform access to the study interventions, all participants were provided a study phone to enable access to the app. Additional modifications made to the app since our study have improved performance of the app on low-end devices, enabling future implementation studies that leverage personal phones or tablet devices. However, not all phones can run virtual simulations.

## Conclusions

For health care professionals providing newborn care in a resource-scarce setting, findings from this cohort study suggest that mobile virtual simulation is sufficient to support retention of essential newborn care knowledge and skills among nurses and midwives during the 6 months following in-person training when used at least monthly. Virtual ENC simulations were acceptable to health professionals and feasible for implementation in a low-resource setting. More clinical and implementation research is needed to explore the impact of virtual simulations on health professionals’ clinical practices and neonatal outcomes.

## Notes

Supplement 1. **eTable 1.** Study Procedures **eTable 2.** Performance on Pre- and Post-Training Assessments Across Demographic Groups **eTable 3.** Participant Feedback on the 6-Month Follow-up Survey

Supplement 2.
Data Sharing Statement
