# Supplementary material for: Essential Newborn Care Virtual Simulations for Skills Retention in Newborn Care
Source: JAMA Netw Open. 2025 Feb 20;8(2):e2460565. doi: 10.1001/jamanetworkopen.2024.60565 (PMC11843367; doi:10.1001/jamanetworkopen.2024.60565)
Supplement: Supplement 1. — eTable 1. Study Procedures eTable 2. Performance on Pre- and Post-Training Assessments Across Demographic Groups eTable 3. Participant Feedback on the 6-Month Follow-up Survey [file jamanetwopen-e2460565-s001.pdf]

Supplementary Online Content

Umoren RA, Ezeaka C, Berkelhamer SK, et al. Essential newborn care virtual simulations for skills retention in newborn care. *JAMA Netw Open*. 2025;8(2):e2460565.
doi:10.1001/jamanetworkopen.2024.60565

eTable 1. Study Procedures

eTable 2. Performance on Pre- and Post-Training Assessments Across Demographic Groups

eTable 3. Participant Feedback on the 6-Month Follow-up Survey

This supplementary material has been provided by the authors to give readers additional information about their work.

© 2025 Umoren RA et al. *JAMA Network Open*.

eTable 1. *Study Procedures*

Study Observations	Before in-person ENC training	After in-person ENC training	Follow-up period (with vENC)
	Baseline	Immediate post course	6 months post course
Educational observations	ENC1/ENC2 KC, BMV, ENC1/ENC2 CSA	ENC1/ENC2 KC, BMV, ENC1/ENC2 CSA ENC1/ENC2 CSB	ENC1/ENC2 KC, BMV, ENC1/ENC2 CSA ENC1/ENC2 CSB

Legend: *vENC: virtual essential newborn care; KC: Knowledge check (multiple-choice questions); BMV: bag and mask ventilation skills checklist; CS: case scenario*

© 2025 Umoren RA et al. *JAMA Network Open*.

eTable 2. *Performance* on Pre- and Post-Training Assessments Across Demographic Groups*

	ENC1 Knowledge Check						Bag and Mask Ventilation Skills						ENC1 Case Scenario A						ENC1 Case Scenario B			
	Pre-Course		Immediate Post-Course		6 Months Post-Course		Pre-Course		Immediate Post-Course		6 Months Post-Course		Pre-Course		Immediate Post-Course		6 Months Post-Course		Immediate Post-Course		6 Months Post-Course	
**Group**	**Median (IQR)**	**P**	**Median (IQR)**	**P**	**Median (IQR)**	**P**	**Median (IQR)**	**P**	**Median (IQR)**	**P**	**Median (IQR)**	**P**	**Median (IQR)**	**P**	**Median (IQR)**	**P**	**Median (IQR)**	**P**	**Median (IQR)**	**P**	**Median (IQR)**	**P**
Profession		0.067		**0.048**		0.45		0.20		0.73		0.31		0.45		0.13		0.71		0.077		0.48
Nurse	75 (70-85)		95 (90-100)†		85 (81-90)		50 (21-64)		93 (86-100)		100 (86-100)		39 (28-50)		67 (61-78)		78 (72-78)		76 (68-84)		88 (76-88)	
Midwife	80 (75-90)		95 (80-95)†		90 (80-95)		57 (29-71)		93 (86-100)		100 (93-100)		39 (28-50)		78 (67-83)		78 (72-83)		80 (72-88)		88 (80-92)	
Level of Practice		0.73		0.64		0.89		**0.008**		0.70		0.21		0.094		0.14		0.18		0.92		0.84
Primary	80 (75-85)		95 (90-98)		85 (80-95)		43 (29-61)		93 (86-100)		96 (86-100)		33 (19-44)		67 (58-78)		78 (78-83)		80 (68-90)		88 (72-92)	
Secondary	80 (72-82)		95 (85-98)		90 (85-91)		57 (29-64)		93 (86-100)		96 (93-100)		39 (31-42)		72 (64-81)		75 (68-78)		76 (70-84)		88 (84-91)	
Tertiary	80 (75-86)		95 (90-100)		85 (85-95)		68 (57-73)		93 (86-100)		100 (98-100)		44 (32-56)		78 (67-79)		78 (72-83)		76 (72-84)		84 (78-90)	
Experience		**0.023**		0.80		0.72		**0.024**		0.53		0.66		0.18		0.40		0.96		0.14		0.28
<5 years	70 (66-80)		95 (91-95)		85 (80-91)		36 (9-57)		86 (80-98)		100 (93-100)		36 (28-39)		78 (68-78)		78 (71-79)		78 (76-87)		92 (86-92)	
5-10 years	80 (75-85)		95 (90-99)		85 (80-95)		43 (29-64)		93 (86-100)		100 (86-100)		33 (22-49)		67 (61-78)		78 (72-79)		76 (68-84)		88 (76-90)	
11-20 years	85 (80-90)		95 (91-100)		90 (85-95)		64 (57-71)		93 (88-100)		100 (91-100)		42 (35-53)		72 (67-78)		78 (71-83)		76 (69-80)		88 (84-90)	
>20 years	80 (79-88)		95 (90-100)		90 (85-95)		61 (55-73)		93 (89-100)		100 (93-100)		47 (33-57)		78 (67-83)		78 (72-83)		86 (78-89)		78 (75-89)	

*Performance expressed as the percentage of possible points for each assessment
†While nurses and midwives had the same median at the immediate post-course timepoint (95%), the ENC1 knowledge check scores of nurses were more often higher than midwives (mean: 95% vs. 92%; IQR: 90-100% vs. 85-95%).

© 2025 Umoren RA et al. *JAMA Network Open*.

eTable 3. *Participant Feedback on the 6-Month Follow-up Survey*

Feedback	N (%)
Rating of the vENC virtual reality program (4 or 5 on 5-point scale*)	
Easy to access	37/57 (65%)
Easy to navigate	36/60 (60%)
Realistic	45/60 (75%)
Pleasing graphics	45/60 (75%)
Provided valuable feedback	51/61 (84%)
A convenient educational resource	50/61 (82%)
Valuable for clinical practice	51/60 (85%)
I would use the vENC virtual reality program again in the future	46/58 (79%)
I would recommend the vENC virtual reality program to my colleagues	47/55 (85%)
Challenges encountered when using the vENC virtual reality program	
I was able to open the digital resource(s), but the navigation was slow	28/62 (45%)
The digital resource(s) froze or crashed after I opened them	8/62 (13%)
I felt nauseous, or developed a headache, when I used the digital resource(s)	17/62 (27%)
I didn't have time to utilize the digital resources	7/62 (11%)
The digital resources are not relevant to my clinical practice	2/62 (3%)
I forgot to carry my phone with me	4/62 (6%)
I carried my phone with me, but I forgot to use it to access the digital resources	7/62 (11%)
I did not remember that I was supposed to use the digital resources	1/62 (2%)
Other challenges not listed above	5/62 (8%)

**The 5-point scale was strongly disagree (1), disagree (2), neutral (3), agree (4), strongly agree (5).*

© 2025 Umoren RA et al. *JAMA Network Open*.
