# Supplementary material for: Essential Newborn Care Virtual Simulations for Skills Retention in Newborn Care
Source: JAMA Netw Open. 2025 Feb 20;8(2):e2460565. doi: 10.1001/jamanetworkopen.2024.60565 (PMC11843367; doi:10.1001/jamanetworkopen.2024.60565)
Supplement: Supplement 2. — Data Sharing Statement [file jamanetwopen-e2460565-s002.pdf]

Data Sharing Statement

Umoren. Essential Newborn Care Virtual Simulations for Skills Retention in Newborn Care. *JAMA Netw Open*. Published February 20, 2025. doi:10.1001/jamanetworkopen.2024.60565

Data
Data available: Yes
Data types: Deidentified participant data
How to access data: Data are available on request to rumoren@uw.edu
When available: With publication

Supporting Documents
Document types: None

Additional Information
Who can access the data: Researchers whose proposed use of the data has been approved.
Types of analyses: For any purpose.
Mechanisms of data availability: With a signed data access agreement after approval of a proposal.
Any additional restrictions: None
